# Revitalizing Dementia Care: Empowering Lives Through Personalized Exercise and Advanced Technologies

**DOI:** 10.3390/healthcare13182294

**Published:** 2025-09-13

**Authors:** Anamarija Kejžar, Vlado Dimovski, Francesco Miele, Vojko Strojnik, Katri Maria Turunen, Simon Colnar

**Affiliations:** 1 Faculty of Social Work/CC UL, University of Ljubljana, SI-1000 Ljubljana, Slovenia; 2 School of Economics and Business & CC UL, University of Ljubljana, SI-1000 Ljubljana, Slovenia; vlado.dimovski@ef.uni-lj.si (V.D.); simon.colnar@ef.uni-lj.si (S.C.); 3 Department of Political and Social Sciences, University of Trieste, 34139 Trieste, Italy; francesco.miele@dispes.units.it; 4 Faculty of Sports & CC UL, University of Ljubljana, SI-1000 Ljubljana, Slovenia; vojko.strojnik@fs.uni-lj.si; 5 Seinäjoki University of Applied Sciences, 60101 Seinäjoki, Finland; katri.turunen@seamk.fi

**Keywords:** exercise, artificial intelligence, technology, information, dementia, patient care planning

## Abstract

**Background/Objectives**: The known benefits of sport and exercise for people with dementia (PwD) and their caregivers mean that physical activity could be prioritized over pharmacological treatment. Research suggests that physical activity not only enhances the overall wellbeing of PwD, but also improves the relationships and wellbeing of their caregivers. The text examines the importance of physical activity for PwD and explores whether certain types of exercise, as well as modern tools like information and communication technology (ICT) and artificial intelligence (AI), are particularly suitable for this population given their different living environments, such as at home or in institutions. **Methods**: The study employed a qualitative design, conducting three focus groups (N = 17) in Slovenia with three distinct participant groups: informal caregivers (N = 6), physiotherapists in care homes (N = 7), and people diagnosed with dementia (N = 4). Data collection involved structured focus group discussions guided by key questions on types of exercise, challenges faced, and potential ICT and AI applications. Descriptive statistics including frequencies, means and standard deviations were used to summarize demographic data of respondents. Given the qualitative nature of the focus groups the emphasis was on thematic content analysis to identify common themes and insights supported by descriptive summaries to contextualize the findings. **Results**: The results suggest that regular physical activity tailored to an individual’s existing lifestyle and abilities can be essential for improving the quality of life of PwD. Although ICT and AI play an important role in promoting and monitoring regular physical activity and a sense of safety, the use of ICT and AI tools are still the exception, not the rule. Key barriers include inadequate awareness of existing solutions, cognitive decline, physical limitations, safety concerns, and limited access to appropriate programs. The study highlights the unused potential of ICT and AI for overcoming these barriers and offers solutions like personalized exercise—which refers to a physical activity program that is tailored to an individual’s specific needs, abilities, preferences, and goals—tracking, adaptive programs, and AI-driven virtual assistants that promote safety and encourage regular physical activity.

## 1. Introduction

Dementia is a progressive neurological syndrome characterised by a decline in cognitive function—including memory, reasoning, language and the ability to perform everyday activities—that significantly impairs daily life. More than 55 million people worldwide live with dementia, and nearly 10 million new cases are reported each year, making it one of the leading causes of disability and care dependency in older adults [1]. The condition places a significant burden not only on healthcare systems, but also on informal carers, who often experience emotional, physical and financial stress [2].

Physical activity is increasingly recognized as a fundamental component of non-pharmacological dementia care [3], yet its value for person with dementia (PwD) is often underappreciated. This may stem from a persistent misconception that PwD have limited rehabilitation potential and are less likely to benefit from physical activity interventions [4]. This viewpoint, however, often reflects an outdated understanding of rehabilitation, which traditionally focused solely on improving functional capacity [5]. In the context of a progressive and complex condition like dementia, the benefits of physical activity should be evaluated more broadly, considering its role in maintaining functional abilities, slowing decline, enhancing quality of life, and fostering a sense of meaning [6]. Regular physical activity not only improves memory and attention performance in PwD but promotes general mental and physical health as well [7]. Furthermore, the social dimension of physical activity offers significant advantages, facilitating social interaction, fostering sociability, and enabling communication, thereby contributing to the wellbeing of PwD by alleviating associated biological and psychological symptoms and, consequently, also supporting caregiver wellbeing [8]. Best results seem to be achieved with multimodal training that combines aerobic, strength, flexibility, and balance exercises [9,10].

Given the heightened risk of falls in PwD, multi-component exercise focusing on muscle strength and balance is also crucial [11], although specific parameters for strength training in this population are still being defined. Recognizing the unique nature of each individual’s condition to make plan for personalized exercise underscores the importance of tailoring rehabilitation approaches, including physical activity, to their specific needs [4]. It is important fo make a personalized plan for exercise for PwD with regard to wishes and, stage of dementia, knowledge and capabilities of carers and possibilities to use ICT/AI in regular exercise of PwD. However, several factors can affect participation and adherence to these programs, including significant cognitive impairments that hinder the ability to follow instructions and remember routines, as well as behavioral symptoms like agitation [12,13], where use of AI/ICT plays also tole of monitoring to increase safety and regular adaptation regarding stage of dementia. Entirely self-directed interventions may not be appropriate, even in the early stages of dementia [14], highlighting the importance of support networks. The social aspect of physical activity also plays an important role in maintaining self-identity [15] and promoting social inclusion within the community [16] to empower lives of PwD and also lives of their carers in home environment.

Also in care home settings, recommendations emphasize multi-component exercise, including strength, endurance, and balance training, ideally performed under the supervision of trained professionals [17]. Research indicates that care home residents with dementia can participate in functional exercise with positive impacts on physical performance [16,18], and they often find such supervised group training enjoyable and safe [19]. However, access to such interventions in care homes is often limited and inequitable compared to residents without dementia [20]. The availability of physical activity interventions by trained providers in care homes varies and is generally quite limited, with only 10–67% of residents having access to physiotherapy [21]. For PwD in care homes, integrating light physical activity into daily routines is crucial and often more feasible than structured exercise programs; however, successful implementation faces barriers such as limited staff awareness, negative attitudes, and resource constraints, which can be exacerbated by staff shortages, hindering the consistent provision of individualized support and person-centered care essential for promoting engagement and positive outcomes [22].

Recent literature highlights the potential of digital technologies including AI to address existing barriers to physical activity and challenges in maintaining engagement for PwD, while also supporting caregivers in monitoring and safety [12].

Several reviews recently assessed the use and impact of these technologies [12,23,24,25]. Cote et al. [24] in a review of 48 clinical studies, highlight the contribution of wearable devices to improving our understanding of the impact of dementia on physical health, showing that PwD generally have lower levels of daily physical activity and sleep efficiency. Other reviews also stress the role of digital tools beyond just monitoring; these devices are increasingly seen as valuable resources to promote physical activity. Dove and colleagues [23] analyzed 31 studies of exergames that engage PwD in intuitive, everyday activities. These activities provide an accessible and enjoyable way for PwD to engage in physical activity while promoting cognitive skills. The authors emphasize the clinical potential of such technologies, but also note the need for strategies to integrate them into everyday environments, educate users, and remove common barriers to physical activity in PwD. In another review, Lee et al. [25] examined 33 quantitative studies that primarily concentrated on wearable devices like wristbands, actigraphy, and accelerometers and their effects on various clinical outcomes, including motor function, mobility, blood pressure, caregiver burden, and behavioral and psychological symptoms. Similarly, in 2024 Sortino et al. [12] confirm these findings in their review of seven studies largely focused on wearable technologies, exergames, and virtual or augmented reality for people with Alzheimer’s disease. However, they note a clear lack of consistency in the exercise protocols that utilize these technologies and call for future research aimed at determining the optimal frequency, intensity, and functional guidelines to maximize the benefits of technology-enhanced physical activities for individuals with dementia.

**Figure 1:**
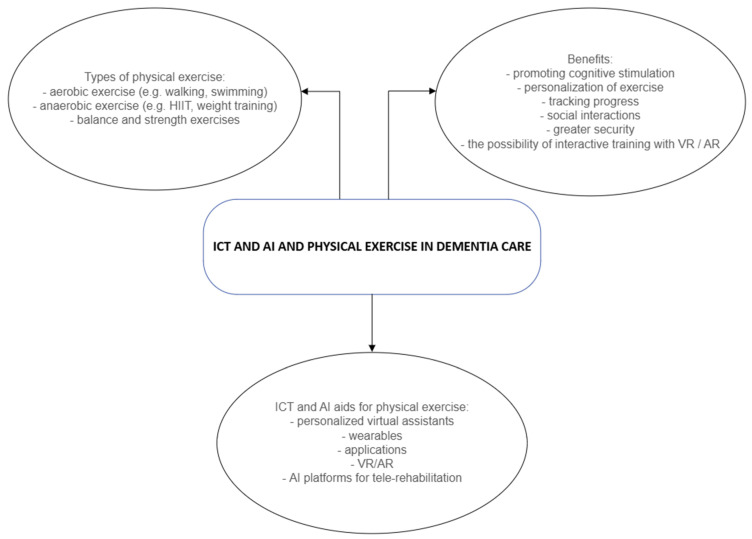
ICT and AI in physical exercise of people with dementia.

In recent years, since AI has experienced a renaissance in healthcare only a limited number of empirical studies have focused on the application of AI to support physical activity, especially in relation to PwD, which can be considered as a gap in existing research. AI-driven systems, based on consolidated technologies such as wearable devices and motion sensors, enable real-time monitoring of physical activity levels and automatic adjustment of activity suggestions based on individuals’ abilities and responses. The use of smart technologies has several advantages: in addition to encouraging regular exercise, it also enables greater safety, location monitoring, body functions and interactive training (see Figure 1). This growing body of evidence calls for a more nuanced understanding of the possibilities of digital tools to support PwD in maintaining an active lifestyle to counteract the cognitive and physical challenges associated with dementia. Integrating personalized exercise, accompanied with the support of smart technologies in daily life of PwD could become a valuable step forward in dementia care by making physical activity more accessible, engaging, and sustainable for PwD while providing caregivers with important monitoring and support tools.

Evidence is limited when it comes to suggested benefits of physical exercise on improved cognition, mobility and functional ability for PwD, which in the opinion of Brett et al. [26] calls for further research. With our study we therefore respond to calls for studies that address gaps in physical exercise characteristics for PwD, which are in our study including frequency of exercise, duration, intensity and type of exercise in different living arrangements [26,27]. Authors Brett et al. [28] explore the effects of exercise on physical performance of PwD, however they warn on cautious interpretation of research findings due to small sample size, which calls for additional insight. In line with this research the intended contribution of our study is to shed light on the topic of exercise and different aspects of physical performance of PwD. While a growing body of evidence highlights the potential of digital tools to support an active lifestyle for PwD [12,23,24,25], most studies adopt a clinical, quantitative approach, focusing on the impact of these technologies rather than the processes of integrating them into daily life. Therefore, this article’s intended contribution is relying on qualitative methods, aiming to explore the cultural, infrastructural, and organizational barriers encountered by PwD and their caregivers in integrating physical activities, while simultaneously investigating their expectations and attitudes towards digital technologies and AI. This research seeks to address the existing gap in understanding how the meanings associated with technologies can either facilitate or hinder their use in dementia care.

## 2. Materials and Methods

The study employed a qualitative, exploratory case study approach to examine the implementation and experience of regular physical activity (PA) with the possible support of ICT and AI among PwD, their informal caregivers, and health professionals. The study was approved by the Management Committee of DEOS (approval number 01/2024) on 17 September 2024. All participants provided informed consent prior to participation. The research was conducted following relevant local legislation and institutional guidelines.

Participants were purposively sampled to capture diverse perspectives, selecting informal caregivers, physiotherapists, and people diagnosed with early-stage dementia to provide comprehensive insight into physical activity practices across home and institutional settings. We included participants with a verified clinical diagnosis of early-stage (first and second stage) dementia, with severity assessed via medical opinion, ensuring they had sufficient communication abilities to participate meaningfully. People with more advanced stages of dementia were excluded from the focus group. Baseline digital literacy was considered informally during recruitment to ensure basic familiarity with the topics of AI and ICT. The final sample included four PwD, six informal caregivers, and seven professionals (Table 1).

We conducted three focus groups (n = 17) to investigate the topic of physical activity in relation to PwD. The research took place from June to October 2024 in Slovenia. The first focus group involved seven physiotherapists. The second focus group involved six relatives (in the role of informal caregivers) of PwD. Our third focus group was conducted with four people diagnosed in the early stage of dementia, and still living independently. The number and size of focus groups were determined based on practical considerations and real-life limitations of the researchers and the aim to capture diverse perspectives across key stakeholder groups. Data were collected through semi-structured interviews, participant observation during exercise sessions, and field notes. Interview guides focused on perceived usability, engagement, benefits, challenges, and broader contextual factors influencing the use of ICT and AI in dementia care with a set of predetermined questions. Semi-structured focus group guides were developed based on a review of the literature on physical activity and dementia care, as well as consultation with experts in dementia care and ICT. The guides included open-ended questions around types and frequency of physical activity, barriers and facilitators experienced by participants, and perceptions of ICT and AI use for supporting physical activity. Separate guides were tailored for each participant group to reflect their unique experiences. An example question for the focus group with physiotherapists is “How much time per day is allocated to physical exercise for residents with dementia?” For the focus group with informal caregivers an example of a question is: “How does exercise take place for a relative with dementia?” In the focus group with PwD we asked them: “What effects of physical exercise do you observe on your mental and physical health?” The rationale for tailoring these guides was to ensure that questions resonated with each group’s experiences and responsibilities, enabling rich, relevant data collection while respecting participants’ cognitive and communication abilities

Focus groups were facilitated by researchers who followed standardized procedures to encourage equitable participation and openness in qualitative research. Each session lasted approximately 90 min, was audio-recorded with participant consent, and held in a private, comfortable setting to maintain confidentiality. Facilitators in some cases used follow-up questions to explore emerging themes while allowing flexible participant-driven conversation. Saturation occurred when no new substantial themes emerged in the focus group discussions. Participant observation and field notes were conducted during a regular physical exercise session only in one care home where some of the physiotherapists work and PwD reside. The observation took place in one session, lasting approximately 45 min. During the session, researchers systematically noted participant engagement, interaction dynamics and behavioral responses that influenced physical activity participation.

The initial coding was carried out by three independent researchers to ensure reliability, using an inductive approach to identify emerging patterns and extracted themes that emerged from our conversations. Disagreements were resolved through discussion until consensus was reached to enhance the reliability of the thematic coding. Researchers compared codes, discussed discrepancies, and consolidated them into a preliminary codebook grouped under broader themes aligning with research questions. In the first phase, the researchers independently repeatedly read the transcripts, while in the second phase we discovered and recorded the most important themes that had emerged during the focus group conversations. NVivo software was used to support the coding and categorization of themes. The analysis did not refer to a specific theoretical framework, which allowed us to keep the research largely exploratory. Data from focus groups, participant observation and field notes were integrated during the thematic analysis process. Themes emerged inductively from the combined data. To assure trustworthiness, the study employed triangulation (multiple data sources and participant types), member checking (some focus group participants were invited to comment on preliminary findings, leading to minor theme adjustments), and peer debriefing (regular internal team discussions challenging assumptions and interpretations) within the research team thus helping to identify and reduce bias and strengthen analytic rigor. Verbatim quotations are provided as illustrative examples that capture key themes emerging across focus groups. Where possible, we indicate the relative prevalence of these views with terms to highlight the salience of particular perspectives. These quotations are intended to deepen understanding of thematic findings rather than to quantify participant responses.

Limitations related to the small sample size and exploratory nature of the study are acknowledged in the discussion section.

Table 2 consists of an example codebook snapshot on key themes such as personalized exercise, digital technology use and quality of life.

Qualitative approaches lend themselves to rich, complex, and under-researched concepts [12], such as the field of physical activity for PwD. We applied our qualitative approach to allow the study participants to describe, interpret, and help us understand their human experiences, which goes hand in hand with the richness of individual experiences [29,30]. By gaining insight into people’s individual experiences through their words and stories, as researchers we were able to gain an insider’s perspective [31] on a very relevant social issue. The overarching theme of the focus groups was to improve understanding of the general field of physical activity for PwD and some important factors that can impact practice, such as identified challenges and barriers, aspirations for the future, and the role of information and communication technologies and artificial intelligence in increasing the physical activity of PwD. Basic descriptive statistics were used to summarize participant demographics and characteristics as shown in Table 2.

## 3. Results

### 3.1. Role of Physical Exercise for PwD

The initial question considered by the first focus group was centered on what kind of physical activity they do with the residents with dementia in the department where there are PwD and in other departments of the care home, given that PwD are also present in other departments there. Most physiotherapists reported being familiar with the PwD’s life history through relatives and staff, which informs their approach to tailoring exercises. Physiotherapist B mentioned the opinion of many physiotherapists that unfortunately there are many “*disruptive factors and many interruptions to the exercise.*” Physiotherapist D highlighted that he also has an opportunity to provide daily exercise and take a more individualized approach to the person’s exercise once a week. Further, physiotherapist D noted the opinion of several respondents in the physiotherapist group that “*people with dementia often have difficulty following the exercises because they are doing something completely different or just watching.*” In the case the person has been very active in their life, we try to adapt, physiotherapist D explained. However, one general observation based of this focus group is that we often have problems with exercises for PwD. Physiotherapist G mentioned that she often incorporates music in her exercise routine since this has several positive effects on PwD. These results could offer a promising starting point for the role of physical activity as a central component of non-pharmacological care for PwD, which is already in the literature well established.

The second question concerned how much time per day is set aside for physical activity for residents with dementia. Exercises typically lasted between 30 and 60 min daily, led by physiotherapists, occupational therapists, or animators. Individualized sessions occurred less frequently when staffing and time allowed. Many physiotherapists mentioned they include some kind of music or singing. Unfortunately, a number of them re-iterated that this type of exercise is often accompanied by interruptions due to the existing condition of PwD.

The third question was about the exercise options available to residents with dementia. Physiotherapist A stated they have an opportunity to participate in group exercises tailored to their physical abilities. If they are no longer able to participate, they have the option of choosing an individualized approach, which is not done consistently but when there is time. Physiotherapist B mentioned as many other of his colleagues that it is difficult to participate in the group type of exercise since people are “*mostly not able to follow the instructions.*” Physiotherapist C noted when it comes to a person’s preferences, “*we ask them what they would like, but do not get an answer.*” Physiotherapist D explained that it is extremely difficult to motivate those participating, which is why they also sing, as PwD are more comfortable and motivated this way. Both physiotherapist E and physiotherapist F stated that PwD enjoy ball games and other simple sporting activities.

We also discussed physical activity with six relatives and asked about their views on physical activity for PwD. Only one person, relative E, stated she is active in a group of pensioners who do exercises together with PwD. All interviewees in this focus group mentioned that they go for regular walks, but unfortunately this was the only aspect that was in any way related to physical activity. To give a deeper insight, relative A as many other relatives noted: “*If I am honest, we do not pay much attention to exercise because I have a lot of other things to take care of.*” She mentions that the person used to go cycling, but due to his condition she now prefers him to stay at home so she can see what he is doing. Relative B said that they do ball games when their grandchildren come to visit and emphasizes the opinion of many relatives that “*we do not have any form of regular exercise, no one mentioned that this could be useful.*” Worryingly, relative C said: “*I feel like we have to stop going for walks because she becomes unpredictable and can say something that embarrasses me”.* Relative E provided some individual positive insights: “*We go for a walk every day, have a lot of fun there, and talk to each other.*”

In the first section of the third focus group with people diagnosed with early-stage dementia, we gained insight into their physical activity in the past and how they engage in physical activity today. Person A explained that he was once very active as he went hiking and cycling with his wife and also played ball games with the children. Nowadays, the situation is different, as he elaborated the fear that some other PwD share: “*I am afraid to walk or cycle because it has happened to me that I got lost and I do not know where I am anymore and I do not feel safe.* *Now I walk every day,*”. Person B told us that they did not use to be very active, they did not have the time. “*When I retired, I started to walk every day and I still do, I walk on a familiar path and can do it myself, I do not need help yet.*” Similarly, person C was not active: “*Today, I have problems with my knee that even make it difficult for me to walk, so it’s hard to imagine me being physically active,*” she notes. Person D differently explained: “*I still go to the gym regularly, I have had my personal trainer there for many years, and I also play badminton. I always gain some strength with sport, so I will try to stay active for as long as I can.*”

### 3.2. Impact of Physical Exercise for People with Dementia

The next section in focus group one with question four was intended to provide information about how PwD feel after exercise. Physiotherapist A optimistically confirmed that exercise helps individuals feel better, yet at the same time explains we need to be honest and shared the opinion of some other colleagues that “*we do not see a significant change in condition of people with dementia.*” Physiotherapist B agreed: “*As we do not expect progress in most cases, we are more concerned and try to prevent the situation from getting worse.*” Physiotherapist G concluded this section of the focus group on a positive note by saying that physical activity benefits everyone, including PwD, since at least communication improves.

While exploring the potential effects of physical activity, relative A said that it could be beneficial because she has noticed that he is happier and calmer after walks as well as in terms of dementia. Relative B noted that the relative with dementia is very happy and in a good mood while playing ball games, especially when the grandchildren are around. According to relative D, the involvement of a physiotherapist could be beneficial and possibly even slow down the progression of dementia. Relative E also believed that this could slow down the progression of the symptoms. In the case of relative F, the opportunity to exercise benefits both the person with dementia and the relative since it gives them both the opportunity to socialize with other people.

As regarding views on the effects of physical activity in the focus group with PwD, person A stated that he feels better when he can go outside and he also sleeps better. He wishes to continue walking in company, but everyone has their own problems and he does not want to burden others with his. He added his own opinion: “*I really miss it and I miss riding my bike. I was very fit, I could probably still do it if I had company, and I could feel safe because I would know I would not get lost.*” Person B was somewhat skeptical about taking up sports in old age, but mentions that it could be interesting if the sport was organized and someone would motivate her to do it. She mentioned having heard as some other PwD have also heard: “*that physical exercise can slow down the progression of dementia, yes, it would be good (to be physically active).*” She added that she feels better after walks. She concludes that she *would* “ *‘like to try a different kind of exercise, I just do not know how.*” Person D was confident he can continue his exercise program. He has also informed his trainer and friends of his diagnosis so that they can help him if “*I get lost in thought or in reality.*” He continued with an illustrative example, “*My wife is a bit embarrassed from time to time and she would rather I just went for a walk, we disagree on that, I can only hope she understands me.*”

### 3.3. Challenges and Barriers Concerning Physical Exercise for People with Dementia

We also explored the challenges and barriers faced by physiotherapists while performing physical exercises with PwD. Physiotherapist A mentioned they encounter new challenges on a daily basis, while physiotherapist B noted that PwD find it difficult to follow instructions, quickly lose interest, and cannot follow some of the others in the group. Physiotherapist C offered a possible solution that some other physiotherapists also emphasized: “*When we lose their attention, we try to win them back by playing ball games or singing, this has proven to be the best strategy so far.*” Physiotherapists D, E, and F agreed that they have gained a lot of knowledge on this topic over their years of experience but emphasize that they lack staff. Physiotherapist G explained that the biggest challenges are understanding and cooperation from the part of PwD since they suffer from behavioral disorders as well.

In the next section of the focus group with relatives, we spoke again about challenges and barriers associated with physical activity for people with PwD. Relative A again noted as many other relatives “*that she is extremely tired as there is a lot of work to do.*” She added that it would be good if someone could give them advice on this topic because they have no prior knowledge. Relative B believed that many options exist, especially as he used to be active, playing soccer with friends and cycling. But “*now I am really worried about what might happen to him, what if he gets injured or lost?*” Relative C was afraid of what this disease will bring. She mentioned that she used to swim in her previous life, but now as several other relatives “*I do not want to take her to the swimming pool because she might behave strangely.*” She stressed “*that people do not know what dementia actually is.*” Relative D said that she would really welcome help with doing different physical activities with PwD. She felt she has almost no energy left.

### 3.4. Can ICT and AI Help with Physical Exercise for PwD?

Our enquiry into the extremely important topics of ICT and AI brought us surprising and generally negative results. Physiotherapist A explained that during the coronavirus pandemic they were doing video calls and online physical exercises. They also tried using virtual glasses, but the residents with dementia had considerable problems with them. The same situation is seen with smartphones, whose usage is low, and people also tend to have difficulties with any kind of video-conferencing technology. Physiotherapists B, C, and D agreed that they do not use information and communication technology solutions as they are unsuitable for their residents. They all emphasized the importance of personal relationships and face-to-face contact for PwD. Physiotherapist B stated the opinion of many physiotherapists as well as informal caregivers and PwD that the residents “are *very eager to have real contact with people, personal contact means a lot to them and, at the end of the day, to make them smile and make their day a little brighter.*” Physiotherapist F said as several other physiotherapists that “*we are dealing with similar disabilities, new things can confuse them, they do not like change and need a lot of time to get used to change.*” In terms of the amount of physical activity, physiotherapist G indicated that this is not the best solution for her residents as they need personal contact and at the same time communicates the orientation towards classically performed exercise—in a group or individually. Unfortunately, none of the physiotherapists had tried adapted exercise for residents with dementia with the help of ICT or AI.

### 3.5. Wishes for the Future Regarding Physical Exercise and AI and ICT Tools for PwD

The last question in the focus group with relatives concerned the area of wishes when it comes to physical activity for PwD in the future. Relative A similarly to other relatives said: *If I was convinced that this would really help, I would also take the time for physical exercise. At the moment, I am not aware of any ICT solutions that could help.”* Relative B noted as several other relatives that “*we would welcome professional support, maybe someone to show us the exercises, maybe the opportunity for group exercises and feedback on whether we are doing things right*.” She also did not know of any devices that could help and expressed her curiosity as to whether anything already exists. Relative D would include her in an exercise group if that would help. “*Is there an exercise program for people with dementia? I do not know if ICT can help, are there any solutions?”* added relative D similarly to others. Relative E would welcome an exercise plan with the help of a physiotherapist and his support. Relative E continued as many other relatives: “*I am unaware of what help ICT tools can offer.*” For relative F, “*since the diagnosis we only solve the most urgent problems*”; “*I am not aware of any tools that are available*”; and “*I would welcome professional help so I know if I am doing the right thing.*” Finally, relative F stated as several other relatives: “*You know, I do everything I can.*”

While discussing future wishes for this important area in the focus group with PwD, person A indicated he would like professional help and guidance. In relation to ICT and AI, he was not sure how it could help him and does not know which options are currently available. Similarly, person B commented: “*Maybe someone could come to our house and help me, perhaps a physiotherapist, who could help me keep my balance and prevent falls.*” Person D really wished to continue with his activities: “*I feel great when I do some kind of physical activity, especially in nature.”* On the subject of technology, he added: “*I know about smartwatches and smartphones, I measure my sleep, my pulse and the number of steps I take, but nothing more. It would be great if someone could give me some advice, at the moment it all depends on me, my wife, and my friends.*”

These findings reflect the diverse aspirations and challenges faced by PwD and their caregivers and provide a foundation for developing more inclusive and responsive dementia-friendly communities.

## 4. Discussion

The World Health Organization defined empowerment as a process through which people gain greater control over decisions and actions affecting their health [32], while the concept of empowerment for PwD is “a confidence-building process whereby people living with dementia are respected, have a voice and are heard, are involved in making decisions about their lives and have the opportunity to create change through access to appropriate resources” [33,34]. For someone who has been active all their life, regular physical activity may mean spending parts of the day doing activities they are familiar with and feel comfortable doing. The diagnosis itself should not limit the ability to exercise in the garden or on the street in the neighborhood. The person with dementia can maintain the sense of personal identity, have a choice and control in his/her life and do what he/she can and want as long as possible.

While a substantial body of literature on physical activity (PA) in dementia care, the use of digital tools and artificial intelligence (AI), and barriers to implementation can already be found, our study: shows the barriers in carers perception about importance of personalized regular exercise, lack of use of ICT/AI—in both home and institutional environment. Despite the small sample size, the study provides qualitative insights into how much attention is given to daily exercise—with or without AI and ICT. ICT and AI-supported PA programs are perceived and experienced by PwD and their formal and informal caregivers. Our findings are consistent with Lewis et al. [6], that any activity is better than none and brings benefits. Our findings are partially consistent with the existing literature on relatives’ limited awareness of the benefits of physical activity, limited understanding of dementia generally, fatigue, lack of motivation, and fear of injury [22,35] and stigma of dementia. The results show there is still not clear understanding of the role of regular and personalized exercise for PwD to wm empower lives of PwD due to readiness, acceptability and lack of different contextual barriers, like knowledge or time. The results didn’t confirm the integral role that physical activity should have in terms of maintaining functional ability, slowing its decline, and contributing to the overall quality of life and meaningfulness [35]. Therefore, our research doesn’t support the overarching efforts to promote physical activity as a basic and fundamental human need [3] for PwD. In the study, we confirmed the theoretical and researched the practical implications of the importance of physical activity for the physical and mental wellbeing of PwD and pointed out the untapped potential of using AI and ICT for maintaining physical activity in the lives of PwD. Our study shows that there is a big gap in using ICT/AI devices to support exercise in daily life of PwD. Both—PwD and informal caregivers confirmed that physical activity (e.g., regular walks) improves the wellbeing of a relative with dementia and thus their own wellbeing. The authors of the article believe that the use of ICT and AI in the area of everyday physical activity for PwD holds great potential from multiple points of view: on one hand, the devices can promote different forms of activity, also enable them to connect with others, share ideas, while, on the other, they allow for the activity of an individual person with dementia to be monitored, which can assist with monitoring and medically treating the progression of dementia. The research demonstrates the necessity of achieving one of the goals of the Dementia Management Strategy in Slovenia by 2030: the development of ICT support for more independent living of PwD [36].

In contrast to previous research often conducted in more controlled or theoretical contexts, our study reflects the challenges and opportunities concerning real-world implementation in home and institutional settings. Another interesting insight from our study is the contradictory finding that some of PwD are actually not able to participate in physical activities, contrary to the research of Telenius et al. [37] and Toots et al. [18]. Importantly, the findings additionally highlight specific facilitators and obstacles related to personalization, usability, and the emotional and relational dimensions of ICT- and AI-based interventions—dimensions that are underexplored in the literature. By adopting a transdisciplinary and participatory approach, the study also proposes a practical framework that can inform the future scaling and refinement of such programs, especially in age-friendly community contexts. The results of all three focus groups conducted allow us to conclude that the promotion and use of physical activity of PwD with the help of ICT and AI remains underutilized, which is consistent with the findings of Ries [4].

We find that there are many barriers to planning personalized exercises for PwD that need to be overcome before we can plan exercises tailored to a person with dementia with the support of ICT/AI. The barriers we discovered in the interviews are the lack of time and awareness of the importance of regular exercise, knowledge and accessibility of different ICT/AI devices, the presence of stigma, ensuring maximum safety from exercise, which causes PwD to be limited in their exercise, which affects the lower quality of life of a person with dementia.

A weakness of the study is that the number of participants in the focus groups was quite limited, which limits the overall generalizability of the findings. However, the purposive sampling strategy enabled the inclusion of diverse stakeholder perspectives directly relevant for our explored topics. Additionally, methodological rigor was maintained through in-depth thematic analysis and researcher checking, which together enhance the credibility and richness of study results despite the limited sample. Another limitation is that the study was conducted in one country only. Nevertheless, the three focus group interviews provided enough diverse data to analyze the views of professionals, relatives, and PwDs on physical activity. A larger number of participants would most likely have been able to provide even more nuanced findings. Yet, since the findings correspond well with earlier studies on physical activity among PwD, however would benefit from additional empirical research. The authors recommend future research considers a wider area (such as European countries), which could provide support for a greater emphasis on physical activity in the care of PwD and the use of ICT and AI in promoting physical activity in dementia care.

One advantage of the research we conducted is the involvement of people with dementia in the early stages, when they can still make their own decisions about their future lives and indicate what they want for the coming years.

As a conclusion, this study shows that caregiver support is critical for promoting the physical activity of PwD, especially as the disease progresses. Unfortunately, caregivers often lack the ability to provide support, may be embarrassed by the signs of dementia, or be burdened by the caregiving situation and lack the resources to promote physical activity. It also seems that rehabilitation professionals require more training to better understand dementia and the cognitive impairments it causes, together with its impact on a person’s functioning and wellbeing. It still appears that rehabilitation is only seen as improving functioning, whereas with progressive diseases like dementia the focus is on maintaining functioning and especially quality of life. Unfortunately, the use of IT and AI tools remains underutilized in both home and institutional settings.

In the study, by comparing regular exercise for PwD in home and institutional settings, we found that concerns about safety and basic care (such as nutrition and personal hygiene) predominated—regular exercise as one of the non-pharmacological activities that could positively contribute to the well-being of PwD can be completely overlooked. As study shows, many residents are limited to moving between the bed in their room and the seat at the dining table in the dining room. Even PwD living at home do not always have the opportunity to go for walks or engage in other activities in the community, as relatives may be embarrassed by possible inappropriate words or behaviour.

## 5. Conclusions

The development of a dementia-friendly environment can only be achieved through the acceptance of people with disabilities and knowledge about dementia. Unfortunately, in Slovenia we find only minimal use of smart technologies in the field of dementia care, which is what we want to draw attention to with the study and promote the use of smart technologies in the life and care of people with dementia. The use of ICT and artificial intelligence has so many benefits—it can encourage regular activity, connect to various online networks, enable monitoring of exercise and vital signs—which can also be a great help to GPs—and it can help carers to reduce the anxiety—“What if it gets lost?”—that keeps many people with dementia trapped in their home or institutional environment. Formal and informal carers have the opportunity to use smart technologies to improve the quality of life of PwD—one of the opportunities is a study that is one of the first in Slovenia on the use of smart technologies in the exercise of PwD.

This study highlights the importance of regular physical activity as an important element of daily life for PwD, which bring multidimensional benefits for PwD and their caregivers. By applying a qualitative and user-centred approach, we have uncovered both the potential and limitations of integrating ICT and AI into the daily physical activity routines of PwD. Our findings suggest that while awareness of the benefits of physical activity is increasing in home and in institutions. In home environments there are many challenges that relatives are facing, such as significant barriers remain—from cognitive limitations and safety concerns, taking care only of basic needs of PwD to a lack of digital literacy and professional guidance. The study confirms that personalized physical activity interventions—especially those supported by empathetic interpersonal relationships—can improve physical and mental well-being, maintain functional abilities and promote autonomy for PwD. In addition, the potential of digital technologies and artificial intelligence to support personalized activity planning, monitoring and motivation is promising but underutilized—in home and institutional environment. Efforts to overcome technological scepticism, improve accessibility and build trust in digital technologies among both caregivers and PwD are crucial for future implementation. Overburdened with daily housework and care, fear for the safety of the PwD, the presence of stigma and consequently shame in the face of inappropriate behavior or communication of the PwD in social contacts outside the home. In the institutional environment, however, we perceive a different obstacle, as physiotherapists do not believe that regular exercise PwD inwD, and because they are sometimes disruptive in group exercises, they can be completely ignored. It is true that there will probably be no progress in terms of movement after regular exercise, but since the positive effects of regular exercise on the physical and mental well-being of PwD have been proven, they should also be regularly included in various forms of exercise adapted to them. Except for the period during COVID-19, when exercise also took place remotely, physiotherapists do not use various forms of smart technology to encourage exercise for residents with dementia, as they believe that personal monitoring, a lot of encouragement and short breaks, which are enriched with singing, are necessary. An analysis of the use of smart technologies in focus groups of informal caregivers and physiotherapists demonstrates that there is great potential and a need for greater use of smart technologies in Slovenia. Since there are few providers of adapted technologies for PwD, only some use smart technologies (primarily smart watches) in the home environment. In this, the awareness of relatives plays a major role, with whom they can plan their daily lives, which also includes regular exercise tailored to the PwD.

The authors recommend further transdisciplinary research and pilot projects in different settings to optimize and to promote the integration of technology-enabled physical activity programs. The authors propose pilot projects in which the usability and acceptability of specific smart technological aids (e.g., success of implementation, monitoring of status and activities, response to prompts, days of continuous use) are tested both at home and in an institutional setting, such as a care home. In pilot projects, adapted protocols could be compared with usual activities (compliance with instructions, safety events, carer burden and selected quality of life sub-areas). In this way, insights could be gained that underpin possible practical recommendations. The project activities and the use of smart technologies could be used as a comparison at the level of physical activity programs in care homes (in Slovenia and beyond, e.g., in the Adrion region). In this way, contextual factors that influence the success or failure of personalized training for PwD, using ICT/AI, could be identified. Involving PwD in the early stages of dementia is crucial to jointly develop meaningful interventions that respect their preferences and support their independence. Ultimately, the successful implementation of ICT- and AI-enabled physical activity in dementia care requires not only technological solutions, but also sustained attention to human relationships, ethical considerations and contextual realities to maintain the quality of everyday life—of PwD and their caregivers.

## Figures and Tables

**Table 1:** Focus group participants: demographic data.

FOCUS GROUP	GENDER	AGE	Mean AGE	SD AGE
Physiotherapists (N = 7)	6 females 1 male	20–30 = 2 30–40 = 2 40–50 = 2	35	8.16
Relatives (N = 6)	5 females 1 male	60–70 = 6	65	0
Persons with dementia (N = 4)	2. females 2 males	60–70 = 2 70–80 = 2	70	5

**Table 2:** Example Codebook Snapshot.

Theme	Code	Definition	Example
Personalized Exercise	Individual Preferences	Tailoring or adapting exercise to individual history or abilities	“We try to adapt the exercises if they were active in the past” (Physiotherapist D)
Digital Technology Use	ICT Awareness	Mentions of knowledge or lack of knowledge regarding ICT solutions	“We are not aware that we could help ourselves with ICT” (Relative E)
Quality of Life	Emotional Benefits	Descriptions of improved mood, wellbeing, social interaction associated with physical activity	“He is happier and calmer after walks” (Relative A)

## Data Availability

The datasets presented in this article are available from AK upon request: anamarija.kejzar@fsd.uni-lj.si.
